# Supplementary material for: Assessing intra-lab precision and inter-lab repeatability of outgrowth assays of HIV-1 latent reservoir size
Source: PLoS Comput Biol. 2019 Apr 12;15(4):e1006849. doi: 10.1371/journal.pcbi.1006849 (PMC6481870; doi:10.1371/journal.pcbi.1006849)
Supplement: S2 Table — (PDF) [file pcbi.1006849.s002.pdf]

Assay	Starting material	Replicates	Dilutions	Stimulation	Target cells	Culture length (days)	Readout	Readout assay
UCSD [1, 6, 22]	100ml whole blood, $7 - 20 \times 10^6$ $rCD4^+$	6	3 fold: 10^6 to 1,372 $rCD4^+$	Plate-bound αCD3/αCD28	MOLT-4 / CCR5	9 – 16	gag	RT-PCR
U. Pitt. [3]	180ml whole blood, 300×10^6 PBMC	6 (5 for smallest cell input)	3 fold: 10^6 to 10^4 $rCD4^+$	PHA + γ-irradiated PBMC	$CD8^-$ PBMC	14	p24	Perkin Elmer HIV-1 p24
JHU [2, 3, 11, 13]	$\geq$ 100 ml whole blood, 400×10^6 PBMC	2 of cell inputs $< 10^6$, 13 – 67 of cell input 10^6 (determined by available material)	5 fold: 10^6 to 320 $rCD4^+$	PHA + γ-irradiated PBMC	MOLT-4 / CCR5	21	p24	Perkin Elmer HIV-1 p24
SR	$\geq$ 100ml whole blood, 400×10^6 PBMC	2 of cell inputs $< 10^6$, 5 – 22 of cell input 10^6 (determined by available material)	5 fold: 10^6 to 320 $rCD4^+$	PHA + γ-irradiated PBMC	$CD8^-$ PBMC	8 – 20	p24	Perkin Elmer HIV-1 p24
