# Supplementary material for: Assessing intra-lab precision and inter-lab repeatability of outgrowth assays of HIV-1 latent reservoir size
Source: PLoS Comput Biol. 2019 Apr 12;15(4):e1006849. doi: 10.1371/journal.pcbi.1006849 (PMC6481870; doi:10.1371/journal.pcbi.1006849)
Supplement: S5 Table — (PDF) [file pcbi.1006849.s005.pdf]

Parameter	Scenario	Median [2.5, 97.5%ile] bias of estimate ($\log_{10}$)	95% CI coverage
σ_a	No excess variation	+0.106 [+0.064, +0.244]	N/A
	Moderate variation (normal-distributed)	+0.038 [−0.017, +0.206]	98.1%
	Moderate variation (gamma-distributed)	+0.037 [−0.017, +0.212]	97.9%
	Large variation (normal-distributed)	+0.014 [−0.194, +0.322]	97.4%
	Large variation (gamma-distributed)	+0.035 [−0.188, +0.406]	96.0%
σ_b	No excess variation	+0.132 [+0.084, +0.283]	N/A
	Moderate variation (normal-distributed)	+0.074 [+0.001, +0.268]	99.8%
	Moderate variation (gamma-distributed)	+0.076 [+0.006, +0.278]	99.5%
	Large variation (normal-distributed)	+0.041 [−0.159, +0.508]	99.3%
	Large variation (gamma-distributed)	+0.056 [−0.152, +0.528]	99.6%
