# Supplementary material for: Assessing intra-lab precision and inter-lab repeatability of outgrowth assays of HIV-1 latent reservoir size
Source: PLoS Comput Biol. 2019 Apr 12;15(4):e1006849. doi: 10.1371/journal.pcbi.1006849 (PMC6481870; doi:10.1371/journal.pcbi.1006849)
Supplement: S6 Table — (PDF) [file pcbi.1006849.s006.pdf]

Parameter	Median [$2.5, 97.5\%$ile] bias of estimate ($\log_{10}$)	Median absolute error of estimate ($\log_{10}$)	95% CI coverage
Total variation $\left(\sqrt{\sigma_a^2+\sigma_b^2+\sigma_c^2}\right)$	$+0.014$ [$-0.095, +0.144$]	0.043	95.1%
Aliquot & batch variation $\left(\sqrt{\sigma_a^2+\sigma_b^2}\right)$	-0.018 [$-0.256, +0.142$]	0.059	97.2%
Aliquot & lab variation $\left(\sqrt{\sigma_a^2+\sigma_c^2}\right)$	$+0.063$ [$-0.043, +0.178$]	0.064	77.9%
Batch & lab variation $\left(\sqrt{\sigma_b^2+\sigma_c^2}\right)$	-0.017 [$-0.238, +0.143$]	0.065	95.6%
σ_a	$+0.045$ [$-0.175, +0.168$]	0.083	88.3%
σ_b	-0.205 [$-0.248, +0.109$]	0.205	91.8%
σ_c	$+0.014$ [$-0.189, +0.194$]	0.115	93.5%
$\beta_{\mathbf{S}}$	$+0.001$ [$-0.279, +0.266$]	0.095	95.0%
β_2	$+0.013$ [$-0.332, +0.367$]	0.126	93.8%
β_3	$+0.001$ [$-0.393, +0.411$]	0.135	94.0%
β_4	-0.014 [$-0.463, +0.407$]	0.143	94.2%
