# Supplementary material for: Assessing intra-lab precision and inter-lab repeatability of outgrowth assays of HIV-1 latent reservoir size
Source: PLoS Comput Biol. 2019 Apr 12;15(4):e1006849. doi: 10.1371/journal.pcbi.1006849 (PMC6481870; doi:10.1371/journal.pcbi.1006849)
Supplement: S7 Table — (PDF) [file pcbi.1006849.s007.pdf]

Parameter	Median [2.5, 97.5%ile] bias of estimate ($\log_{10}$)	Median absolute error of estimate ($\log_{10}$)	95% CI coverage
Total variation $\left(\sqrt{\sigma_a^2 + \sigma_b^2 + \sigma_c^2}\right)$	+0.016 [−0.092, +0.149]	0.044	94.8%
Aliquot & batch variation $\left(\sqrt{\sigma_a^2 + \sigma_b^2}\right)$	−0.016 [−0.259, +0.146]	0.059	97.3%
Aliquot & lab variation $\left(\sqrt{\sigma_a^2 + \sigma_c^2}\right)$	+0.064 [−0.042, +0.179]	0.065	76.9%
Batch & lab variation $\left(\sqrt{\sigma_b^2 + \sigma_c^2}\right)$	−0.015 [−0.234, +0.148]	0.065	95.4%
σ_a	+0.044 [−0.175, +0.174]	0.083	88.7%
σ_b	−0.203 [−0.248, +0.114]	0.203	92.2%
σ_c	+0.014 [−0.189, +0.196]	0.119	93.4%
$\beta_{\mathbf{S}}$	+0.000 [−0.282, +0.268]	0.095	95.3%
β_2	+0.013 [−0.330, +0.367]	0.127	93.7%
β_3	+0.001 [−0.396, +0.408]	0.134	93.9%
β_4	−0.012 [−0.469, +0.415]	0.142	94.3%
