# Supplementary material for: Assessing intra-lab precision and inter-lab repeatability of outgrowth assays of HIV-1 latent reservoir size
Source: PLoS Comput Biol. 2019 Apr 12;15(4):e1006849. doi: 10.1371/journal.pcbi.1006849 (PMC6481870; doi:10.1371/journal.pcbi.1006849)
Supplement: S8 Table — (PDF) [file pcbi.1006849.s008.pdf]

Parameter	Median [2.5, 97.5%ile] bias of estimate ($\log_{10}$)	Median absolute error of estimate ($\log_{10}$)	95% CI coverage
Total variation $\left(\sqrt{\sigma_a^2 + \sigma_b^2 + \sigma_c^2}\right)$	$+0.017$ $[-0.094, +0.149]$	0.044	94.7%
Aliquot & batch variation $\left(\sqrt{\sigma_a^2 + \sigma_b^2}\right)$	-0.016 $[-0.255, +0.145]$	0.060	97.3%
Aliquot & lab variation $\left(\sqrt{\sigma_a^2 + \sigma_c^2}\right)$	$+0.064$ $[-0.041, +0.181]$	0.066	76.4%
Batch & lab variation $\left(\sqrt{\sigma_b^2 + \sigma_c^2}\right)$	-0.013 $[-0.235, +0.150]$	0.064	95.6%
σ_a	$+0.047$ $[-0.175, +0.173]$	0.085	88.5%
σ_b	-0.206 $[-0.248, +0.116]$	0.207	92.1%
σ_c	$+0.008$ $[-0.189, +0.198]$	0.118	93.7%
$\beta_{\mathbf{S}}$	$+0.000$ $[-0.281, +0.269]$	0.096	95.5%
β_2	$+0.014$ $[-0.331, +0.369]$	0.126	93.6%
β_3	$+0.001$ $[-0.393, +0.411]$	0.135	94.1%
β_4	-0.014 $[-0.464, +0.411]$	0.142	94.5%
