# Supplementary material for: Assessing intra-lab precision and inter-lab repeatability of outgrowth assays of HIV-1 latent reservoir size
Source: PLoS Comput Biol. 2019 Apr 12;15(4):e1006849. doi: 10.1371/journal.pcbi.1006849 (PMC6481870; doi:10.1371/journal.pcbi.1006849)
Supplement: S9 Table — (PDF) [file pcbi.1006849.s009.pdf]

| Condition | Probability of misidentifying source of variation (median and 95% CI) | | | Performance of total variation estimate $\left(\sqrt{\sigma_a^2+\sigma_b^2+\sigma_c^2}\right)$ | | |
|---|---|---|---|---|---|---|
| | Source | Posterior prob. that parameter $> 0$ | Posterior prob. that parameter $\geq$ median estimate in Table 4 | Median $[2.5, 97.5\%\text{ile}]$ bias ($\log_{10}$) | Median absolute error ($\log_{10}$) | 95% CI coverage |
| $\sigma_a = 0$ | $\sigma_a$ | 34.9% (19.4% - 94.5%) | 2.3% (0.2% - 66.0%) | $+0.005$ $[-0.119, +0.119]$ | 0.034 | 95% |
| $\sigma_b = 0$ | $\sigma_b$ | 76.3% (35.0% - 99.9%) | 19.6% (0.6% - 88.7%) | $+0.010$ $[-0.077, +0.150]$ | 0.040 | 93% |
| $\sigma_c = 0$ | $\sigma_c$ | 63.7% (31.8% - 99.5%) | 37.0% (3.5% - 98.7%) | $+0.002$ $[-0.104, +0.083]$ | 0.031 | 96% |
| $\sigma_a = \sigma_b = 0$ | $\sigma_a$<br>$\sigma_b$ | 33.8% (20.0% - 79.5%)<br>99.4% (55.9% - 100.0%) | 0.1% (0.0% - 6.9%)<br>24.7% (0.6% - 83.4%) | $+0.023$ $[-0.094, +0.139]$ | 0.054 | 91% |
| $\sigma_a = \sigma_c = 0$ | $\sigma_a$<br>$\sigma_c$ | 31.9% (20.6% - 94.7%)<br>99.1% (49.9% - 100.0%) | 1.7% (0.1% - 49.6%)<br>78.2% (5.3% - 99.0%) | $+0.001$ $[-0.091, +0.080]$ | 0.026 | 96% |
| $\sigma_b = \sigma_c = 0$ | $\sigma_b$<br>$\sigma_c$ | 41.8% (26.8% - 73.6%)<br>41.9% (25.6% - 77.4%) | 0.5% (0.0% - 12.6%)<br>1.7% (0.0% - 28.2%) | $+0.023$ $[-0.056, +0.095]$ | 0.032 | 92% |
| $\sigma_a = \sigma_b = \sigma_c = 0$ | $\sigma_a$<br>$\sigma_b$<br>$\sigma_c$ | 32.9% (22.7% - 67.0%)<br>34.0% (23.5% - 78.5%)<br>34.2% (23.1% - 82.5%) | 0.0% (0.0% - 2.0%)<br>0.1% (0.0% - 3.0%)<br>0.0% (0.0% - 4.1%) | $+0.044$ $[+0.015, +0.138]$ | 0.044 | 95% |
