# Supplementary material for: Assessing intra-lab precision and inter-lab repeatability of outgrowth assays of HIV-1 latent reservoir size
Source: PLoS Comput Biol. 2019 Apr 12;15(4):e1006849. doi: 10.1371/journal.pcbi.1006849 (PMC6481870; doi:10.1371/journal.pcbi.1006849)
Supplement: S12 Table — Batch variation-free ensemble estimates of parameters were used in simulations. Each entry shows median improvement of row versus column assay (median and 95% CI sampling from ensemble parameter distribution). IUPMs are shown on the U. Pitt. scale. Darker shading: Entire CI is above zero (green) or below zero (red). Lighter shading: Median estimate is above 0.05 (> 12% increase, green) or below −0.05 (> 11% decrease, red), but the CI crosses zero. “Infinite” difference in accuracy indicates that a majority of simulations of the disfavored assay have all-negative outcomes (maximum likelihood estimate of zero). Assay configurations match those in the experimental study, except “JHU (8M)” and “SR (8M),” which use fewer wells to match the cell input count of U. Pitt. and UCSD assays. (PDF) [file pcbi.1006849.s012.pdf]

Change in $\log_{10}$ error, IUPM = 0.1	U. Pitt.	UCSD	JHU	SR	JHU (8M)	SR (8M)
U. Pitt.		-0.34 (-0.46 to -0.19)	-0.27 (-0.43 to 0.12)	+∞ (-0.32 to +∞)	0.38 (-0.37 to +∞)	+∞ (-0.11 to +∞)
UCSD	0.34 (0.19 to 0.46)		0.06 (-0.04 to 0.40)	+∞ (0.04 to +∞)	0.70 (0.00 to +∞)	+∞ (0.24 to +∞)
JHU	0.27 (-0.12 to 0.43)	-0.06 (-0.40 to 0.04)		+∞ (-0.01 to +∞)	0.59 (-0.02 to +∞)	+∞ (0.11 to +∞)
SR	-∞ (-∞ to 0.32)	-∞ (-∞ to -0.04)	-∞ (-∞ to 0.01)		0.00 (-∞ to +∞)	0.00 (0.00 to +∞)
JHU (8M)	-0.38 (-∞ to 0.37)	-0.70 (-∞ to 0.00)	-0.59 (-∞ to 0.02)	0.00 (-∞ to +∞)		0.00 (-0.13 to +∞)
SR (8M)	-∞ (-∞ to 0.11)	-∞ (-∞ to -0.24)	-∞ (-∞ to -0.11)	0.00 (-∞ to 0.00)	0.00 (-∞ to 0.13)	

Change in $\log_{10}$ error, IUPM = 0.2	U. Pitt.	UCSD	JHU	SR	JHU (8M)	SR (8M)
U. Pitt.		-0.05 (-0.09 to 0.00)	-0.02 (-0.08 to 0.11)	0.18 (-0.06 to +∞)	0.06 (-0.08 to +∞)	+∞ (-0.04 to +∞)
UCSD	0.05 (0.00 to 0.09)		0.03 (-0.03 to 0.14)	0.23 (-0.02 to +∞)	0.10 (-0.03 to +∞)	+∞ (0.01 to +∞)
JHU	0.02 (-0.11 to 0.08)	-0.03 (-0.14 to 0.03)		0.19 (-0.05 to +∞)	0.09 (-0.06 to +∞)	+∞ (-0.01 to +∞)
SR	-0.18 (-∞ to 0.06)	-0.23 (-∞ to 0.02)	-0.19 (-∞ to 0.05)		-0.08 (-∞ to +∞)	0.19 (-0.10 to +∞)
JHU (8M)	-0.06 (-∞ to 0.08)	-0.10 (-∞ to 0.03)	-0.09 (-∞ to 0.06)	0.08 (-∞ to +∞)		0.46 (-0.15 to +∞)
SR (8M)	-∞ (-∞ to 0.04)	-∞ (-∞ to -0.01)	-∞ (-∞ to 0.01)	-0.19 (-∞ to 0.10)	-0.46 (-∞ to 0.15)	
