# Supplementary material for: Assessing intra-lab precision and inter-lab repeatability of outgrowth assays of HIV-1 latent reservoir size
Source: PLoS Comput Biol. 2019 Apr 12;15(4):e1006849. doi: 10.1371/journal.pcbi.1006849 (PMC6481870; doi:10.1371/journal.pcbi.1006849)
Supplement: S13 Table — Method and format match S12 Table. (PDF) [file pcbi.1006849.s013.pdf]

| Change in $\log_{10}$ error, IUPM = 0.5 | U. Pitt. | UCSD | JHU | SR | JHU (8M) | SR (8M) |
|---|---|---|---|---|---|---|
| U. Pitt. | | -0.06 (-0.09 to 0.00) | -0.05 (-0.10 to 0.02) | 0.01 (-0.08 to 0.34) | 0.00 (-0.08 to 0.15) | 0.04 (-0.09 to +∞) |
| UCSD | 0.06 (0.00 to 0.09) | | 0.00 (-0.03 to 0.06) | 0.07 (-0.02 to 0.39) | 0.05 (-0.02 to 0.19) | 0.10 (-0.02 to +∞) |
| JHU | 0.05 (-0.02 to 0.10) | 0.00 (-0.06 to 0.03) | | 0.06 (-0.03 to 0.40) | 0.05 (-0.03 to 0.19) | 0.09 (-0.03 to +∞) |
| SR | -0.01 (-0.34 to 0.08) | -0.07 (-0.39 to 0.02) | -0.06 (-0.40 to 0.03) | | -0.02 (-0.34 to 0.12) | 0.04 (-0.16 to +∞) |
| JHU (8M) | 0.00 (-0.15 to 0.08) | -0.05 (-0.19 to 0.02) | -0.05 (-0.19 to 0.03) | 0.02 (-0.12 to 0.34) | | 0.05 (-0.12 to +∞) |
| SR (8M) | -0.04 (-∞ to 0.09) | -0.10 (-∞ to 0.02) | -0.09 (-∞ to 0.03) | -0.04 (-∞ to 0.16) | -0.05 (-∞ to 0.12) | |

| Change in $\log_{10}$ error, IUPM = 1 | U. Pitt. | UCSD | JHU | SR | JHU (8M) | SR (8M) |
|---|---|---|---|---|---|---|
| U. Pitt. | | -0.02 (-0.04 to 0.01) | -0.03 (-0.05 to 0.02) | 0.01 (-0.04 to 0.13) | 0.01 (-0.03 to 0.10) | 0.04 (-0.04 to 0.18) |
| UCSD | 0.02 (-0.01 to 0.04) | | -0.01 (-0.03 to 0.03) | 0.03 (-0.02 to 0.16) | 0.03 (-0.01 to 0.12) | 0.06 (-0.02 to 0.20) |
| JHU | 0.03 (-0.02 to 0.05) | 0.01 (-0.03 to 0.03) | | 0.04 (-0.02 to 0.16) | 0.04 (-0.02 to 0.12) | 0.06 (-0.02 to 0.20) |
| SR | -0.01 (-0.13 to 0.04) | -0.03 (-0.16 to 0.02) | -0.04 (-0.16 to 0.02) | | 0.00 (-0.13 to 0.08) | 0.02 (-0.14 to 0.18) |
| JHU (8M) | -0.01 (-0.10 to 0.03) | -0.03 (-0.12 to 0.01) | -0.04 (-0.12 to 0.02) | 0.00 (-0.08 to 0.13) | | 0.02 (-0.08 to 0.17) |
| SR (8M) | -0.04 (-0.18 to 0.04) | -0.06 (-0.20 to 0.02) | -0.06 (-0.20 to 0.02) | -0.02 (-0.18 to 0.14) | -0.02 (-0.17 to 0.08) | |

| Change in $\log_{10}$ error, IUPM = 2 | U. Pitt. | UCSD | JHU | SR | JHU (8M) | SR (8M) |
|---|---|---|---|---|---|---|
| U. Pitt. | | -0.01 (-0.03 to 0.01) | -0.01 (-0.04 to 0.04) | 0.01 (-0.03 to 0.07) | 0.02 (-0.02 to 0.07) | 0.03 (-0.02 to 0.13) |
| UCSD | 0.01 (-0.01 to 0.03) | | -0.01 (-0.03 to 0.05) | 0.02 (-0.02 to 0.08) | 0.03 (-0.02 to 0.08) | 0.04 (-0.02 to 0.13) |
| JHU | 0.01 (-0.04 to 0.04) | 0.01 (-0.05 to 0.03) | | 0.02 (-0.04 to 0.08) | 0.03 (-0.03 to 0.08) | 0.04 (-0.03 to 0.14) |
| SR | -0.01 (-0.07 to 0.03) | -0.02 (-0.08 to 0.02) | -0.02 (-0.08 to 0.04) | | 0.01 (-0.06 to 0.08) | 0.02 (-0.04 to 0.11) |
| JHU (8M) | -0.02 (-0.07 to 0.02) | -0.03 (-0.08 to 0.02) | -0.03 (-0.08 to 0.03) | -0.01 (-0.08 to 0.06) | | 0.01 (-0.06 to 0.11) |
| SR (8M) | -0.03 (-0.13 to 0.02) | -0.04 (-0.13 to 0.02) | -0.04 (-0.14 to 0.03) | -0.02 (-0.11 to 0.04) | -0.01 (-0.11 to 0.06) | |
