# Supplementary material for: Assessing intra-lab precision and inter-lab repeatability of outgrowth assays of HIV-1 latent reservoir size
Source: PLoS Comput Biol. 2019 Apr 12;15(4):e1006849. doi: 10.1371/journal.pcbi.1006849 (PMC6481870; doi:10.1371/journal.pcbi.1006849)
Supplement: S14 Table — Method and format match S12 Table. (PDF) [file pcbi.1006849.s014.pdf]

Change in $\log_{10}$ error, IUPM = 0.1	U. Pitt.	UCSD	JHU	SR	JHU (8M)	SR (8M)
U. Pitt.		-0.36 (-0.53 to -0.20)	-0.37 (-0.73 to -0.01)	+∞ (-0.30 to +∞)	+∞ (-0.55 to +∞)	+∞ (-0.11 to +∞)
UCSD	0.36 (0.20 to 0.53)		-0.02 (-0.23 to 0.28)	+∞ (0.06 to +∞)	+∞ (-0.11 to +∞)	+∞ (0.23 to +∞)
JHU	0.37 (0.01 to 0.73)	0.02 (-0.28 to 0.23)		+∞ (0.03 to +∞)	0.60 (0.00 to +∞)	+∞ (0.18 to +∞)
SR	-∞ (-∞ to 0.30)	-∞ (-∞ to -0.06)	-∞ (-∞ to -0.03)		0.00 (-∞ to +∞)	0.00 (0.00 to +∞)
JHU (8M)	-∞ (-∞ to 0.55)	-∞ (-∞ to 0.11)	-0.60 (-∞ to 0.00)	0.00 (-∞ to +∞)		0.00 (-0.08 to +∞)
SR (8M)	-∞ (-∞ to 0.11)	-∞ (-∞ to -0.23)	-∞ (-∞ to -0.18)	0.00 (-∞ to 0.00)	0.00 (-∞ to 0.08)	

Change in $\log_{10}$ error, IUPM = 0.2	U. Pitt.	UCSD	JHU	SR	JHU (8M)	SR (8M)
U. Pitt.		-0.04 (-0.10 to 0.02)	-0.09 (-0.29 to 0.05)	0.22 (-0.06 to +∞)	-0.01 (-0.23 to +∞)	0.85 (-0.02 to +∞)
UCSD	0.04 (-0.02 to 0.10)		-0.05 (-0.23 to 0.09)	0.26 (-0.02 to +∞)	0.03 (-0.16 to +∞)	0.88 (0.02 to +∞)
JHU	0.09 (-0.05 to 0.29)	0.05 (-0.09 to 0.23)		0.33 (0.01 to +∞)	0.11 (-0.02 to +∞)	1.03 (0.05 to +∞)
SR	-0.22 (-∞ to 0.06)	-0.26 (-∞ to 0.02)	-0.33 (-∞ to -0.01)		-0.19 (-∞ to +∞)	0.20 (-0.07 to +∞)
JHU (8M)	0.01 (-∞ to 0.23)	-0.03 (-∞ to 0.16)	-0.11 (-∞ to 0.02)	0.19 (-∞ to +∞)		0.55 (-0.11 to +∞)
SR (8M)	-0.85 (-∞ to 0.02)	-0.88 (-∞ to -0.02)	-1.03 (-∞ to -0.05)	-0.20 (-∞ to 0.07)	-0.55 (-∞ to 0.11)	
