# Supplementary material for: Assessing intra-lab precision and inter-lab repeatability of outgrowth assays of HIV-1 latent reservoir size
Source: PLoS Comput Biol. 2019 Apr 12;15(4):e1006849. doi: 10.1371/journal.pcbi.1006849 (PMC6481870; doi:10.1371/journal.pcbi.1006849)
Supplement: S15 Table — Method and format match S12 Table. (PDF) [file pcbi.1006849.s015.pdf]

Change in log_{10} error, IUPM = 0.5	U. Pitt.	UCSD	JHU	SR	JHU (8M)	SR (8M)
U. Pitt.		-0.04 (-0.10 to 0.02)	-0.13 (-0.29 to -0.04)	0.02 (-0.08 to 0.41)	-0.06 (-0.23 to 0.08)	0.06 (-0.09 to +∞)
UCSD	0.04 (-0.02 to 0.10)		-0.08 (-0.24 to 0.02)	0.07 (-0.03 to 0.42)	-0.02 (-0.20 to 0.14)	0.10 (-0.03 to +∞)
JHU	0.13 (0.04 to 0.29)	0.08 (-0.02 to 0.24)		0.15 (0.03 to 0.61)	0.06 (-0.02 to 0.19)	0.19 (0.02 to +∞)
SR	-0.02 (-0.41 to 0.08)	-0.07 (-0.42 to 0.03)	-0.15 (-0.61 to -0.03)		-0.09 (-0.54 to 0.08)	0.05 (-0.15 to +∞)
JHU (8M)	0.06 (-0.08 to 0.23)	0.02 (-0.14 to 0.20)	-0.06 (-0.19 to 0.02)	0.09 (-0.08 to 0.54)		0.12 (-0.09 to +∞)
SR (8M)	-0.06 (-∞ to 0.09)	-0.10 (-∞ to 0.03)	-0.19 (-∞ to -0.02)	-0.05 (-∞ to 0.15)	-0.12 (-∞ to 0.09)	

Change in log_{10} error, IUPM = 1	U. Pitt.	UCSD	JHU	SR	JHU (8M)	SR (8M)
U. Pitt.		-0.02 (-0.05 to 0.01)	-0.11 (-0.27 to -0.02)	0.02 (-0.05 to 0.13)	-0.06 (-0.23 to 0.05)	0.04 (-0.05 to 0.22)
UCSD	0.02 (-0.01 to 0.05)		-0.09 (-0.26 to 0.00)	0.03 (-0.03 to 0.14)	-0.05 (-0.22 to 0.07)	0.05 (-0.03 to 0.24)
JHU	0.11 (0.02 to 0.27)	0.09 (0.00 to 0.26)		0.13 (0.03 to 0.32)	0.05 (-0.01 to 0.12)	0.15 (0.03 to 0.41)
SR	-0.02 (-0.13 to 0.05)	-0.03 (-0.14 to 0.03)	-0.13 (-0.32 to -0.03)		-0.08 (-0.28 to 0.04)	0.02 (-0.12 to 0.20)
JHU (8M)	0.06 (-0.05 to 0.23)	0.05 (-0.07 to 0.22)	-0.05 (-0.12 to 0.01)	0.08 (-0.04 to 0.28)		0.10 (-0.03 to 0.36)
SR (8M)	-0.04 (-0.22 to 0.05)	-0.05 (-0.24 to 0.03)	-0.15 (-0.41 to -0.03)	-0.02 (-0.20 to 0.12)	-0.10 (-0.36 to 0.03)	

Change in log_{10} error, IUPM = 2	U. Pitt.	UCSD	JHU	SR	JHU (8M)	SR (8M)
U. Pitt.		-0.01 (-0.03 to 0.02)	-0.10 (-0.26 to -0.01)	0.01 (-0.03 to 0.08)	-0.06 (-0.23 to 0.03)	0.03 (-0.02 to 0.12)
UCSD	0.01 (-0.02 to 0.03)		-0.09 (-0.26 to -0.01)	0.02 (-0.03 to 0.09)	-0.05 (-0.22 to 0.04)	0.03 (-0.02 to 0.12)
JHU	0.10 (0.01 to 0.26)	0.09 (0.01 to 0.26)		0.12 (0.01 to 0.30)	0.04 (-0.01 to 0.09)	0.13 (0.03 to 0.32)
SR	-0.01 (-0.08 to 0.03)	-0.02 (-0.09 to 0.03)	-0.12 (-0.30 to -0.01)		-0.07 (-0.26 to 0.03)	0.01 (-0.05 to 0.10)
JHU (8M)	0.06 (-0.03 to 0.23)	0.05 (-0.04 to 0.22)	-0.04 (-0.09 to 0.01)	0.07 (-0.03 to 0.26)		0.09 (-0.02 to 0.28)
SR (8M)	-0.03 (-0.12 to 0.02)	-0.03 (-0.12 to 0.02)	-0.13 (-0.32 to -0.03)	-0.01 (-0.10 to 0.05)	-0.09 (-0.28 to 0.02)	
