# Supplementary material for: Assessing intra-lab precision and inter-lab repeatability of outgrowth assays of HIV-1 latent reservoir size
Source: PLoS Comput Biol. 2019 Apr 12;15(4):e1006849. doi: 10.1371/journal.pcbi.1006849 (PMC6481870; doi:10.1371/journal.pcbi.1006849)
Supplement: S16 Table — (PDF) [file pcbi.1006849.s016.pdf]

	JHU protocol			
	Weaker therapy (3x), $N = 12$ per arm		Stronger therapy (10x), $N = 6$, no control arm	
	Unbatched	Batched	Unbatched	Batched
Median estimated fold-reduction	3.13	3.06	10.67	9.91
Median bias	+4%	+2%	+7%	−1%
Median $\log_{10}$ absolute error	0.10	0.08	0.23	0.21
Accuracy improvement from batching (% reduction in abs. error)	20%		9%	
Coverage of 95% CI	88.4%	87.2%	91.0%	90.4%
Power ($p < 0.05$)	90.5%	98.6%	77.5%	79.2%
Power improvement from batching (percentage point increase)	8%		2%	

	UCSD protocol			
	Weaker therapy (3x), $N = 12$ per arm		Stronger therapy (10x), $N = 6$, no control arm	
	Unbatched	Batched	Unbatched	Batched
Median estimated fold-reduction	3.12	3.03	10.52	9.71
Median bias	+4%	+1%	+5%	−3%
Median $\log_{10}$ absolute error	0.11	0.08	0.21	0.18
Accuracy improvement from batching (% reduction in abs. error)	27%		14%	
Coverage of 95% CI	88.4%	87.3%	90.7%	89.3%
Power ($p < 0.05$)	90.5%	99.4%	83.2%	90.9%
Power improvement from batching (percentage point increase)	9%		8%	
