# Supplementary figures and images for: Assessing intra-lab precision and inter-lab repeatability of outgrowth assays of HIV-1 latent reservoir size
Source: PLoS Comput Biol. 2019 Apr 12;15(4):e1006849. doi: 10.1371/journal.pcbi.1006849 (PMC6481870; doi:10.1371/journal.pcbi.1006849)

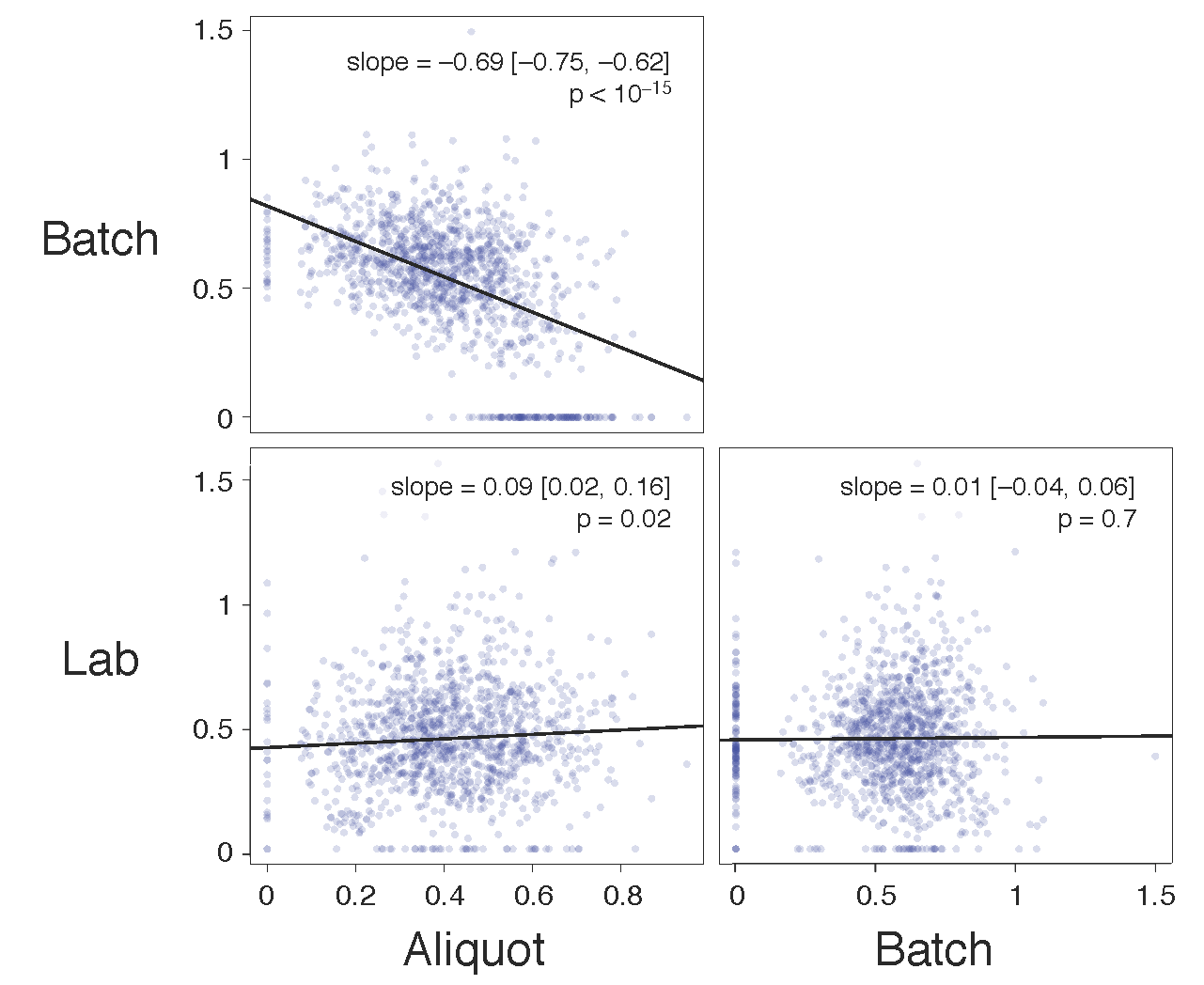

Supplement: S1 Fig — Panels show robust linear regression and Wald test p-values (null model = zero slope) of 1000 samples from the ensemble posterior. (TIF) [file pcbi.1006849.s017.tif]

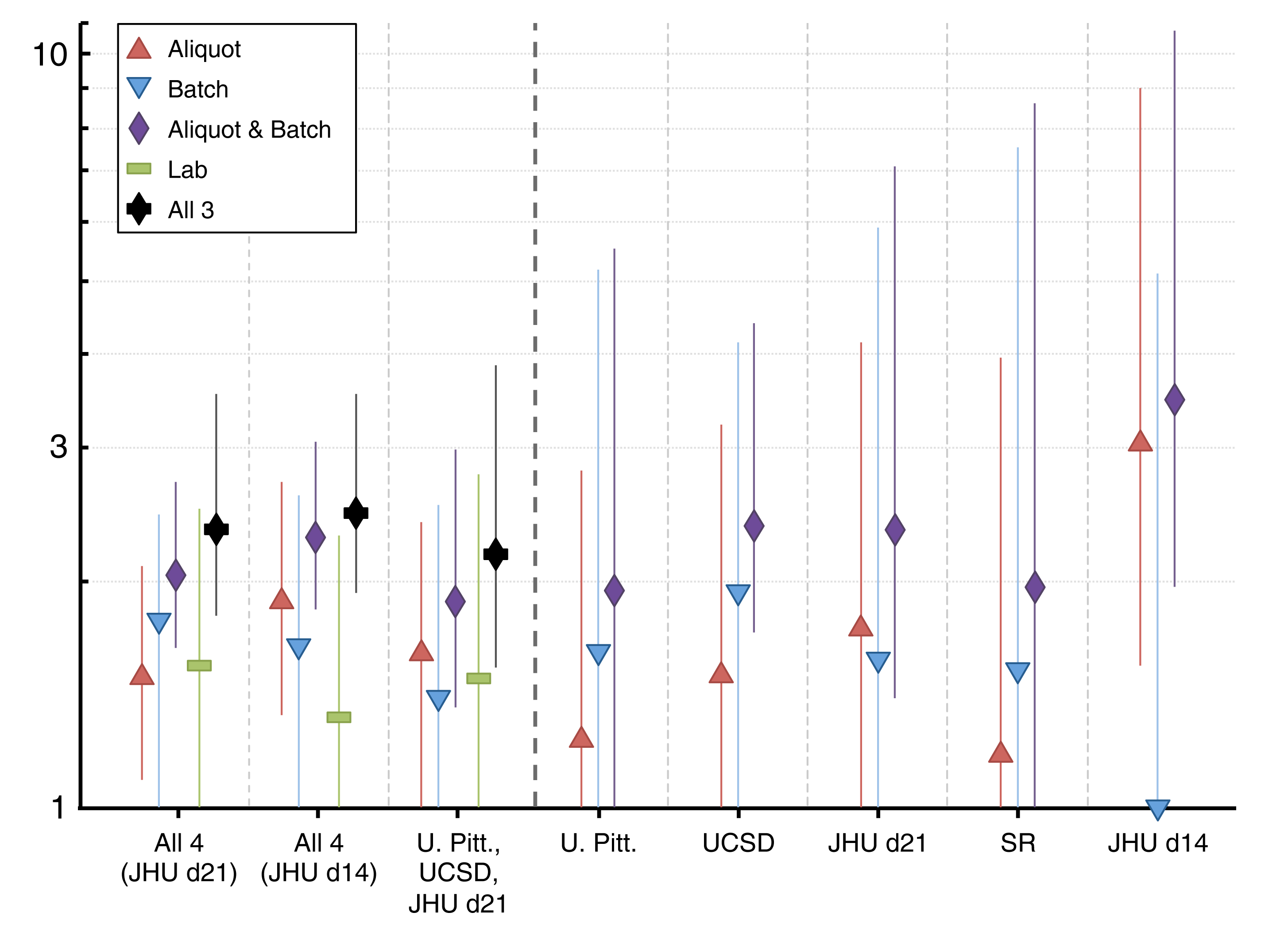

Supplement: S2 Fig — (TIF) [file pcbi.1006849.s018.tif]

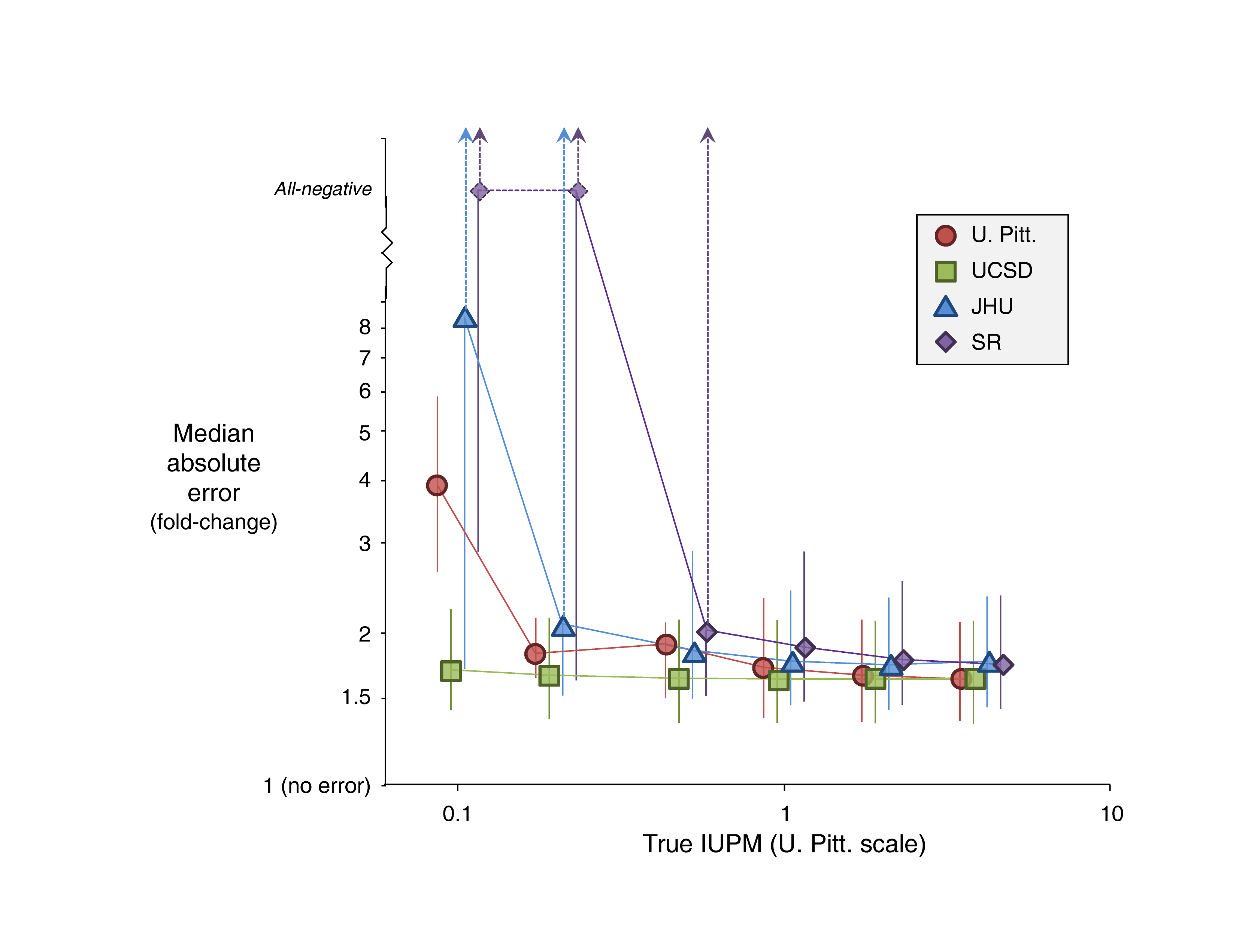

Supplement: S3 Fig — Each assay is measured against a consensus standard, appropriately scaled by βl for that assay. “All-negative” on the y-axis represents infinite error on the fold-change scale, which occurs when the maximum likelihood estimate of IUPM is zero. Bars show 95% credible intervals around median posterior estimates. (TIF) [file pcbi.1006849.s019.tif]
